# Decentralization of hepatitis B care in sub-Saharan Africa: study protocol for a prospective cohort assessing three models of care in rural Ethiopia

**DOI:** 10.1186/s12913-025-13340-1

**Published:** 2025-09-30

**Authors:** Kristian Braathen Malme, Nega Berhe, Fufa Hunduma, Hailemichael Desalegn, Asgeir Johannessen

**Affiliations:** 1 Department of Infectious Diseases, Vestfold Hospital Trust, Tønsberg, Norway; 2 Department of Infectious Diseases, Akershus University Hospital, Lørenskog, Norway; 3 Aklilu Lemma Institute of Pathobiology, Addis Ababa University, Addis Ababa, Ethiopia; 4 Department of Epidemiology, St Paul’s Hospital Millennium Medical College, Addis Ababa, Ethiopia; 5 Toronto Centre for Liver Disease, University Health Network, University of Toronto, Toronto, ON Canada; 6 Medical Department, St Paul’s Hospital Millennium Medical College, Addis Ababa, Ethiopia; 7 Sustainable Health Unit (SUSTAINIT), University of Oslo, Oslo, Norway; 8 Akershus University Hospital, Postboks, Lørenskog, 1000, 1478 Norway

**Keywords:** Hepatitis b virus, Ethiopia, Sub-Saharan africa, Models of care, Decentralization, Implementation science

## Abstract

**Background:**

Improving treatment uptake among people with chronic hepatitis B (CHB) infection who live outside urban centres is crucial to achieving the World Health Organization (WHO) elimination targets. Furthermore, as the number of deaths due to hepatitis B in Africa is rising, there is a need for new models of decentralized care adapted to the local context. The main aim of this study is to compare three different models of decentralized care for CHB treatment in Ethiopia, both from a clinical perspective and an implementation perspective.

**Methods:**

This will be prospective cohort study comparing three models of decentralized care for the management of CHB in Ethiopia. The first model (“standard model”) utilizes the current WHO guidelines for CHB management. The second model (“simplified model”) offers treatment to all individuals with CHB except those with a completely normal baseline assessment, and the third model (“test-and-treat model”) provides treatment to all individuals with CHB.

The study will enroll 4,500 individuals with CHB over a one-year period across six study sites in Ethiopia. All patients will be followed up for 3 years, and the primary outcome is death or liver failure. The three models will be compared using a Cox proportional hazards regression model adjusting for potential confounders.

Additionally, we will design adequate implementation strategies to address barriers and facilitators to implementation. We will design, implement and evaluate a policy toolbox that will ensure local adoption and implementation, using a co-creation approach, bringing together policymakers, politicians, healthcare personnel and patients, to come up with adequate policy implementation strategies for their contexts.

**Discussion:**

From a public health perspective, identifying a simple, feasible and effective model of decentralized CHB care would be a key step to reduce liver-related mortality on the African continent. This study may have important policy implications and is expected to inform national and international hepatitis B elimination efforts in low- and middle income countries (LMICs).

**Trial registration:**

ClinicalTrials.gov, NCT06586983. Registered on September 4, 2024.

## Background

Although the number of people newly infected by chronic hepatitis B (CHB) has declined in recent years, the estimated number of deaths due to CHB is increasing [1]. Over 60% of new hepatitis B infections occur in the World Health Organization (WHO) African region, but only 4% of people living with CHB have been tested in the same region [1]. Furthermore, treatment with Tenofovir disoproxil fumarate (TDF) or other nucleos(t)ide analogues (NA) can effectively prevent liver fibrosis and liver disease [2, 3], but only 0.2% of the estimated population with CHB in the WHO African Region are reached with treatment [1].

As identified by the WHO, a key challenge for LMICs to improve testing and treatment, is decentralization of care [1]. In Ethiopia, hepatitis B treatment and care is only available in larger cities [4, 5], while the majority of the population are living in rural areas [6]. With a population exceeding 100 million, and a hepatitis B prevalence at 6.0% [7], decentralized models of care are needed to reduce liver-related mortality. However, international CHB guidelines may not apply in this setting since modern tools such as hepatitis B virus (HBV) deoxyribonucleic acid (DNA) testing, liver stiffness measurements (LSM) or liver biopsy [8, 9] are unavailable in rural areas of Ethiopia. Previous studies have demonstrated how the lack of these tools can act as barriers to care [10]. Although the WHO has published an updated guideline with a simplified treatment algorithm [11], models of care suited to rural populations in LMICs with limited laboratory services and lack of specialized healthcare personnel are yet to be assessed. If the WHO elimination goals for viral hepatitis are to be reached [12], new models of CHB care tailored to these populations are needed.

In this study, we aim to test and compare three models of care at the primary care level in rural Ethiopia, and to explore and identify the best ways to implement CHB care and treatment in this setting. By testing three different models of care, and assesing both clinical and programmatic outcomes, we aim to identify the model of care best suited to rural poulations in LMICs. Furthermore, we plan to evaluate the implementation process, to identify barriers and facilitators, and to understand how to best implement a complex intervention in a resource-limited setting [13–16].

## Methods

### Study design

This will be prospective cohort study comparing three models of decentralized care for the management of CHB in Ethiopia. The overreaching research question is “What is the best model of care for testing, treatment, and follow-up in a primary hospital/health center in rural Ethiopia”. The three models of care will be assessed both from a clinical, programmatic and implementation perspective.

Testing, screening and linkage to care is common for the three model, but the treatment protocol differs. The standard model utilizes the WHO 2024 treatment guideline for hepatitis B to identify individuals at *high risk* of disease progression («*treat only if.*.») [11]. As most primary hospitals in Ethiopia lack assess to HBV DNA testing, we will use the WHO treatment eligibility criteria for settings without an HBV DNA analysis option (Table 1). The simplified model will utilize clinical and laboratory criteria to identify patients at *low risk* of liver injury, who will be exempted from treatment («*treat all except …*»). Finally, the test-and-treat model will offer treatment to all individuals with CHB (“*treat all*”), mimicking the models of care that have been successfully assessed and implemented for HIV and hepatitis C [17]. This latter model avoids the complicated patient assessment and the demand for expensive laboratory tests.

**Table 1:** The three models of care to be assessed in the study: treatment criteria, laboratory analyses required, and scheduled follow-up

	Standard model “treat only if…”	Simplified model “treat all except…”	Test and treat model “treat all”
Treatment criteria	Treat *only* if:	Treat all, *except* if:	Treat all
	-Clinically diagnosed cirrhosis	-There are no signs, symptoms or risk factors for liver disease, *AND*	
	-APRI above 0.5	-APRI is 0.3 or below	
	-Persistently elevated ALT		
	-Co-infections and co-morbidity		
	Pregnant women: treat to prevent mother-to-child transmission		
Laboratory analysis required at inclusion	AST, ALT, platelets, (Creatinine^1^)	AST, platelets (Creatinine^1^)	(Creatinine^1^)
	HIV, HCV, HBsAg RDTs	HIV, HCV, HBsAg RDTs	HIV, HCV, HBsAg RDTs
Follow-up:on treatment	Physical examination, HIV, and pharmacy refill at 3 months, then every 6 months	Physical examination, HIV, and pharmacy refill at 3 months, then every 6 months	Physical examination, HIV and pharmacy refill at 3 months, then every 6 months
	AST, ALT, platelets, (creatinine^1^), HBsAg, DBS every 12 months	AST, platelets, (creatinine^1^), HBsAg, DBS every 12 months	(Creatinine^1^), HBsAg, DBS every 12 months
Follow-up:*not* on treatment	Physical examinationAST, ALT, plateletsHBsAg,DBSevery 6 months	Physical examinationAST, plateletsHBsAg,DBSevery 6 months	N/A

*Abbreviations: ALT* alanine aminotransferase, *APRI* aspartate aminotransferase to platelets ration, *AST* aspartate aminotransferase, *HCV* hepatitis C virus, *HbsAg* hepatitis B surface antigen, *HIV* human immunodeficiency virus, *DBS* dried blood spot testing, *N/A* Not applicable, *RDT* rapid diagnostic test

^1^ Only required in patients at risk of renal disease, i.e. individuals with hypertension, diabetes, use of nephrotoxic drugs, or age above 60 years

### Objectives

1. *Study clinical efficacy (death*,* liver decompensation) of different models of decentralized CHB care*.
2. *Study programmatic success indicators (linkage to care*,* loss to follow-up) with different models of decentralized CHB care*.
3. *Evaluate the implementation of CHB care (Context*,* process*,* and outcomes) of different models*.
4. *Study laboratory success indicators (suppression of viral load*,* human immunodeficiency virus (HIV) incidence) in different models of decentralized CHB care*.
5. *Compare all outcome indicators (points 1–3 above) of decentralized care with the results of hospital-based care from similar settings*.
6. *Study the effect of community screening for hepatitis B*.

### Study setting

**Fig. 1:**
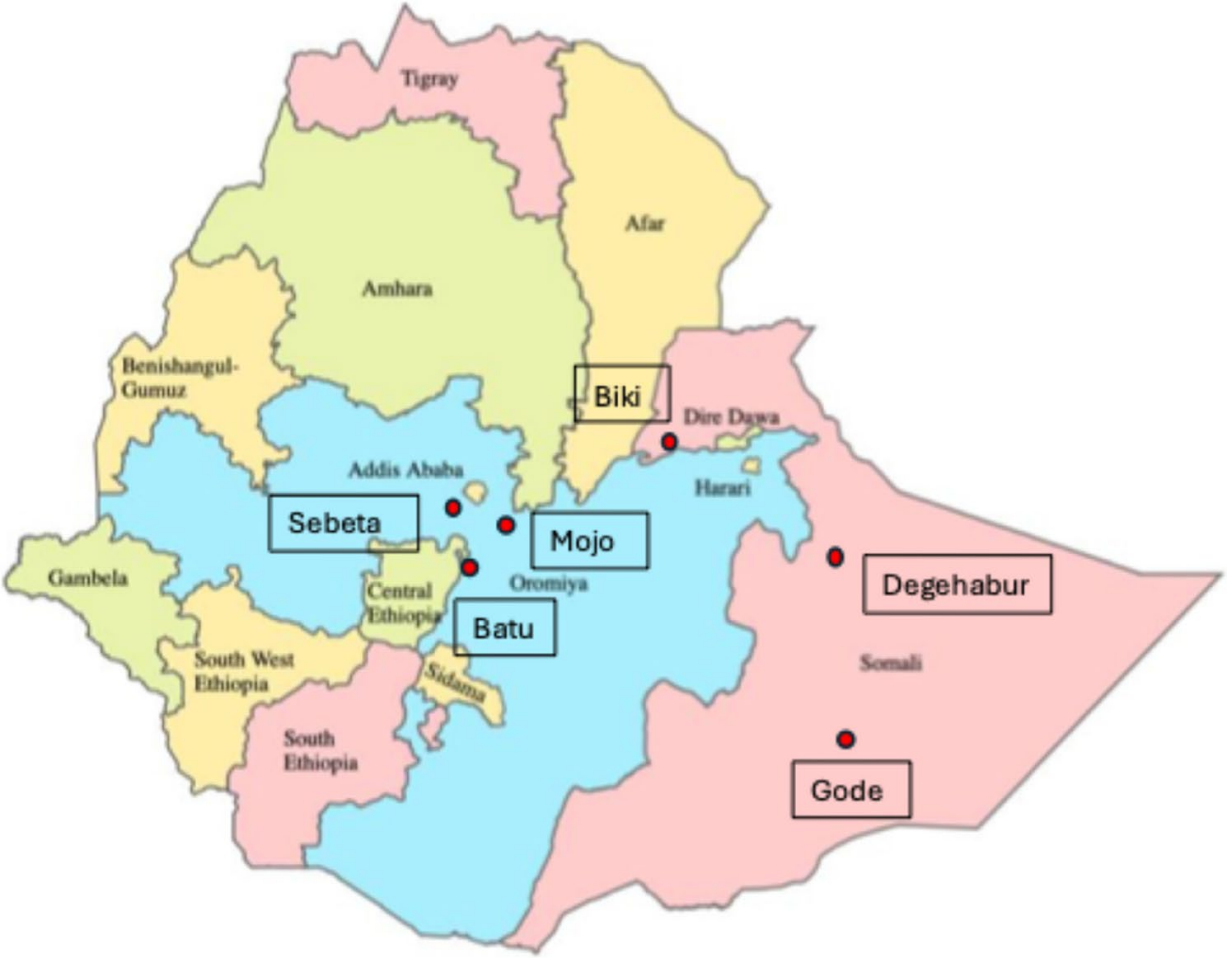
Map of Ethiopia, with the 12 regional states. The study sites are illustrated with red dots. Original image from www.wikipedia.com, reproduced and adapted accordinly to the creative commons attribution-share alike 4.0 international license (https://commons.wikimedia.org/wiki/File: Regions_of_Ethiopia_EN.svg)

The study will be established at six primary hospitals and health centers in rural Ethiopia (Fig. 1), and will leverage on research infrastructure at Addis Ababa University and St Paul’s Hospital Millennium Medical College in Addis Ababa. Three study sites were selected from the Oromia region due to its relative ease of access from Addis Ababa for training of staff, shipment of drugs and equipment, and travel for the study teams. Another three study sites were selected from the Somali region in Ethiopia, which were chosen based on previous collaboration with the local health authorities and the relatively stable geopolitical situation. In total, the Oromia and Somali regional states have > 100 primary hospitals and > 1500 health centers serving a predominantly rural population of approximately 44 million people [18, 19]. Primary hospitals and health centers usually offer outpatient and inpatient services, maternal and child facilities including antenatal care (ANC), emergency surgery, HIV clinics, and access to basic laboratory services such as alanine aminotransferase (ALT), aspartate aminotransferase (AST), hematology (including platelets). A primary hospital typically serve between 60,000 and 100,000 inhabitants.

Two sites will be assigned to the standard model of care («treat only if…»), two sites to the simplified model of care («treat all except…»), and two sites to the test-and-treat model («treat all»). Each model will include one site situated in the Oromia region and one in the Somali region.

The final selection of the study sites were done in close collaboration with the regional health bureaus in the Oromia and Somali regions. As the study aimed to assess care in rural areas, primary hospitals or health centers in rural areas of these regions were considered. Only primary hospitals or health centers having an ART clinic were considered, leveraging the capacity for screening, counselling, initiation of antiviral treatment, long-term follow-up, and tracing efforts in cases of loss to follow-up. Furthermore, sites assigned to the standard or simplified model were required to have access to standard laboratory services such as hematology and biochemistry (ALT, AST) analysis.

Thus, participants will be recruited from the following six sites:

#### Standard model of care

Mojo district hospital (Oromia region) has approximately 80 beds and has an established HIV clinic caring for approximately 250 individuals. The hospital is located 179 km from Addis Ababa, and has a catchment population of approximately 70,000 individuals (Nafyad, Mosisa: Regional database. Oromia Regional Health Bureau, Addis Ababa, Ethiopia, personal communication). The hospital has access to basic laboratory services including biochemistry.

Degehabur hospital (Somali region) is situated in Degehabur town with approximately 48,000 inhabitants [19]. It provides internal medicine, general surgery, paediatrics, gynecology/obstetrics, and has an HIV clinic. This primary hospital has a catchment area of approximately 1,000,000 inhabitants from a vast and scarcely populated area (Mussa, Ahmed: Regional database. Somali Regional Health Bureau Jigjiga, Ethiopia, personal communication).

#### Simplified model of care

Batu general hospital (Oromia region) is located in Batu town, in the East Shewa zone, approximately 160 km from Addis Ababa. The town has approximately 80 000 people [19]. The hospital has 60 beds, and has a well-functioning HIV clinic.

Gode general hospital (Somali region) is located in the Shabelle zone, and has a catchment area of approximately 1,500,000 inhabitants (Mussa, Ahmed: Regional database. Somali Regional Health Bureau Jigjiga, Ethiopia, personal communication). The main in-patient services at the hospital are medical, surgical, pediatrics, orthopedics, gynecology-obstetrics, dermatology, psychiatry, intensive care, and a malnutrition center. The hospital also runs an HIV clinic.

#### Test-and-treat model of care

Sebata health centre (Oromia region) lies in the south west Shewa zone around 40 km from Addis Ababa. The center employs 40 clinical staff (including one physician), and has an HIV clinic providing care to 1200 patients. The catchment area of the health center is approximately 50,000 individuals (Nafyad, Mosisa: Regional database. Oromia Regional Health Bureau, Addis Ababa, Ethiopia, personal communication).

Biki general hospital is located in Biki town with approximately 10,000 inhabitants [19]. The town lies 250 km from Jigjiga, the capital of the Somali region, but with limited access due to poor road conditions. The hospital has a catchment population of approximately 300,000 (Mussa, Ahmed: Regional database. Somali Regional Health Bureau Jigjiga, Ethiopia, personal communication), and has basic laboratory services such as biochemistry and a well-run HIV clinic.

Each study site will have a team composed of 3-5 individuals responsible for screening, inclusion, delivery of the intervention, and follow-up of participants. This will typically be one medical doctor (or health officer), one nurse, one laboratory technician and one data clerk. Team composition will depend on location, accesibility of personnel, and experience of those identified. The study teams will be identified by the local study site and fully integrated into existing clinical infrastructure at the sites. The study aims to utilize existing staff employed at the local health centers/hospitals.

### Sample size and duration of the study

The sample size has been calculated for the primary outcome (death or liver decompensation) with 80% power and 5% risk of type I error. We anticipate that 5% of all included patients (treated and untreated) will either die or experience liver decompensation within 3 years, with the standard model [20], and we want to detect a 50% relative reduction (Odds Ratio of 0.5 or less) with the test-and-treat model. This results in a sample size of 997 patients per arm [21]. We will increase the sample size by 50% and aim for 1,500 patients per model of care arm, due to expected attrition. This results in a total of 4,500 patients at six different sites. Assuming that approximately 9% of those living in the study setting are hepatitis B surface antigen (HBsAg) positive and 50% of those screened can be included in the study, we estimate that we need to screen approximately 100,000 individuals in order to enroll 4,500 CHB patients.

As inclusion rates may vary across sites, we plan to continue inclusion until all models of care have reached 1500 inclusions, accepting an uneven distribution between the two clinics assigned to the model. We expect to complete inclusion within 1 year. As the observation period will be three years, the total study period will be four years. The first participant was enrolled at September 5, 2024, and recruitment is ongoing. 

### Screening activities

Screening for HBsAg at the various sites will be conducted as determined by the local teams, and will include screening at the hospital premises, at local schools, markets, factories, or religious institutions. Rapid diagnostic tests (RDTs) will be used for screening, based on availablility. A confirmatory test will be done at enrolment in the CHB programme. After initial screening, HBsAg positive individuals will counselled and referred to the HBV clinic for HBsAg confirmation testing using the WHO prequalified Determine HBsAg 2 RDT (Abbott Diagnostics, IL, USA) and enrolment in CHB care. All HIV negative adults>18 years with a positive HBsAg confirmation test are eligible for inclusion. Those who are found to be HIV positive at baseline will be referred to the local HIV treatment unit, and not be enrolled in the present project.

### Recruitment and obtaining informed consent

All eligible individuals will be informed about the study in their mother tongue and requested to provide written informed consent prior to study enrollment. If consent is provided, a unique Study ID will be assigned. All included participants will be asked to provide a telephone number where they can be reached (personal or to a relative), for later contact tracing if needed. Participants can withdraw from the study at any given time.

### Eligibility criteria

All adults (>18 years of age) and who are HBsAg positive and provide informed consent are eligible for inclusion. Inclusion and exclusion criteria are given in Table 2.

**Table 2:** Inclusion and exclusion criteria of the study

**Inclusion criteria**	**Exclusion criteria**
Adults (>18 years of age)	Below 18 years of age
HBsAg positive. Informed consent	Negative HBsAg rapid test at screening visit
	HIV positive
	Other disease with short life expectancy (advanced HCC, disseminated cancer, etc.)

*Abbreviations: HbsAg* hepatitis B surface antigen, *HCC* hepatocellular carcinoma, *HIV* human immunodeficiency virus

### Liver disease assessment 

Liver disease assessment will be based upon ALT, aspartate aminotransferase to platelets ratio (APRI) score and clinical assessment (signs of decompensated liver disease) in the standard model, APRI and clinical assessment in the simplified model, and clinical assessment in the test-and-treat model. Laboratory tests at baseline and at follow-up depends on which model of care the study site is assigned to (Table 1). All models will consider radiological signs of liver disease if the participant has undergone imaging the previous 3 years before enrolment, but imaging will not be part of the routine assessment.

### Treatment initiation and follow-up

If a participant is eligible for treatment, pre-treatment counselling will be delivered by a member of the team. Participants will be encouraged to bring a familiy member or friend to the counselling to reduce stigma and promote adherence. Counseling will focus on the natural course of hepatitis B, its long-term consequences, benefits of therapy, possible side effects, the risk of resistance with irregular treatment, and the risk of hepatitis flares if treatment is interrupted. Patients will be warned against excessive alcohol and khat use, herbal medications and over-the-counter drugs. Sex partners and children will be encouraged to be tested, and those without evidence of current or past infection will be advised to get HBV vaccine.

Treatment consists of tenofovir disoproxil fumarate (TDF) 300mg tablets, taken once daily. All patients will be followed up for the duration of the trial, but at various intervals depending on model of care and treatment status (Table 1). Patients with signs of advanced chronic liver disease (ascites, jaundice, confusion, upper GI bleeding) at inclusion will start treatment immediately and get an appointment with a doctor within 2 weeks to evaluate if they need other supportive treatment and care.

At the follow-up appointments the local investigator / study nurse will communicate the previous laboratory results and reiterate what the study entails and pinpoint the responsibilities of the patient. Patients will be asked about adverse events and adherence to therapy. Full clinical examination is not required, but evidence of hepatic decompensation (ascites, jaundice, confusion, upper gastrointestinal bleeding) will be noted.

Patients who do not attend follow-up consultations will be contacted by the team. All participants will be provided with *patient appointment cards* that state their Study ID and the date of their next appointment. If possible, we will send an SMS or call to remind them about the appointment 1 week ahead of time.

Upon inclusion, drugs will be dispensed for 3 months. Once the patient proves to be adherent to therapy, drugs can be dispensed for 6 months by the study team. Empty pill boxes should be brought to each visit for pill count, and the study nurse will register the number of pills dispensed and returned for each follow-up appointment*.*

In individuals without liver cirrhosis, treatment can be stopped if there are two consecutive negative HBsAg RDTs minimum three months apart.

### Pregnant women

Pregnant women will be offered antiviral treatment for their own health if they fulfil the criteria in the designated model. In any case, pregnant women will be given antiviral treatment from gestational week 24-28 to prevent mother-to-child transmission of HBV. This is in addition to recommending three-dose hepatitis B vaccination in all infants, plus timely (within 24 hours) birth dose vaccine. If the mother does not fulfil any of the treatment criteria (individual, for own health), treatment can be stopped once the infant has completed the HBV vaccination series (usually given at 6-10-14 weeks of age). 

All mothers enrolled in the program must be asked to return with their baby at the age 6-12 months for a HBsAg rapid test.

### Management of HCC 

Participants with an abdominal mass suspect of HCC, either at inclusion or at follow-up, will be referred to further diagnostic and therapeutic procedures based on local availability.

### Data collection and management 

Data will be collected prospectively during the study period and entered into an electronic case report form (RedCap) by data clerks at the various sites. Data quaility will be monitored monthly by a central data manager, and missing data/queries will be communicated to the teams on regular intervals. Names, date of birth, contact information and health information are collected from participants. Forms containing participant information will be kept at locked and secure locations at the study sites. Data will be entered in RedCap using study-specific ID numbers, but names, date of birth or other identifiable information will not be entered. No code-list will be used. To ensure confidentiality, only data clerks and members of the project managemnt group has access to the RedCap data set. Only members of the project management group will have access to the final data set.

### Outcomes 

The primary endpoint will be either death or liver decompensation (composite endpoint). Liver decompensation will be detected clinically (ascites, jaundice, encephalopathy, upper gastrointestinal bleeding). Deaths will be verified by health extension workers, who will trace/identify patients WHO miss appointments for more than 3 months. Health extension workers are individuals with a minimum of 10^th^ grade schooling who are part of the Ethiopian public health extension program. Each village has two health extension workers whose main responsibility is to deliver preventative and promotive health services at community level.

Secondary endpoints include programmatic outcomes (linkage to care, retention in care) and laboratory outcomes (viral suppression *viz*HBV DNA ≤10 IU/ml; new HIV infection). HBV DNA is not part of routine care at these sites but will be collected using dried blood spots (DBS) during the study period and measured after completion of the observation period (three years) for those who have received treatment, using the Xpert HBV viral load kit (Cepheid, CA, USA). 

### Implementation research

The implementation objects will be the three different models of CHB care. We will begin by conducting a study of the context where the implementation will take place, and identify context-related barriers and facilitators [22]. Context is defined as a set of characteristics and circumstances that consist of active and unique factors, within which the implementation is embedded [16]. Setting on the other hand is the specific physical location, in which the intervention is put into practice and interacts with context and implementation [16]. For this purpose, we will use the Context and Implementation of Complex Interventions (CICI) [16] and Consolidated Framework for Implementation Research (CFIR) framework as a theoretical guide [23]. Identifying contextual barriers and facilitators of implementation will help to tailor appropriate strategies to mitigate the barriers and cultivate the facilitating factors [16]. We will then evaluate the process of implementation of complex intervention of the CHB treatment and care program. Implementation evaluation provides additional information on how different structures and resources are used, the roles of implementation agents, and the reasoning of various actors [14, 15, 24]. In general, process evaluation is how we can assess the success of implementation strategies, while outcome evaluation evaluates the success of the intervention [25]. In this process evaluation, we will answer the overarching question, ‘What factors influence their implementation process and success?’ We answer this question by assessing important indicators of implementation effectiveness, such as community screening, health education, and linkage to care.

Finally, we will assess implementation outcomes with appropriate implementation science methodologies. Implementation outcomes include acceptability, reach, feasibility, fidelity, adoptability, sustainability, and cost-effectiveness of the program [13, 26].

### Statistical analysis 

Results will be reported using descriptive statistics. Continous data will be reported as mean with standard deviation (SD), and categorical data as number with percentage. Cox proportional hazards regression models will be used to analyze the primary outcome (death and liver decompensation). The primary purpose of the analysis is to assess the association between the model of care and time to event, accounting for potential confounding factors. We will estimate hazard rations (HR), and 95% confidence intervals (CI) for the association between care model type (standard, simplified, test-and-treat) and the outcome. The model will be adjusted for baseline covariates known to be associated with adverse outcome, including age, sex and liver cirrhosis. The proportional hazards assumption will be tested using Schonefeld residuals. Individuals lost to follow-up will be right-censored at the last data known to be alive. Unadjusted survival functions will be estimated using Kaplan-Meier methods and compared using the log-rank test. A sensitivity analysis assuming that all individuals with missing outcome data at the end of the observation period reached the primary outcome (worst case scenario) will be performed.

In a secondary analysis, we will compare outcome variables in the decentralized models with previous results from a centralized model [4]. This comparison will have its inherent limitations due to differences in demography, co-morbidity, and available resources, which we can only partly adjust for in statistical models. However, it will still be of value to be able to assess the decentralized models in a wider context.

STATA (College Station, TX, USA) and/or R Core Team (2024) will be used to analyze the data.

### Oversight, monitoring and dissemination

A compact project management group will organize the training and oversee the implementation of the study. The project management group will visit the study sites at regular intervals throughout the study period to monitor progression, data quality, and confidentiality. A data monitoring committee has not been considered necessary. TDF has a very favorable safety profile. However, spontaneously reported adverse events will be recorded and assessed by the study management team. Additionally, individuals at risk of renal disease (age>60 years, diabetes mellitus, hypertension) will be subjected to regular monitoring of renal function (s-creatinine). There is no interim analysis planned, and no stopping procedure prepared. This is an observational trial and no external audits are planned. However, internal audits by the study management team will be performed at each study site at least annually. A data analysis plan and plan for dissemination will be prepared before data extraction has been completed. Results will be communicated to participants, stakeholders, and healthcare professionals. Abstracts will be submitted to scientific conferences and results published in peer-reviewed scientific journals.

Authorship will be determined according to the ICMJE authorship criteria. Contributors who meet all four criteria will be listed as authors on resulting publications. No professional medical writers will be used.

## Discussion

Here, we provide the structure and details of a prospective, multicenter cohort study assessing decentralized hepatitis B care in Ethiopia, aiming to compare three distinct models of care. We will assess clinical and programmatic outcomes over a 3-year period, and utilize methods from implementation science to assess the context where CHB treatment and care will be implemented. The study may have implications for patients with CHB as well as national and international health authorities and stakeholders, as the number of deaths due to CHB in the WHO Africa region is increasing and countries in the same region are struggling to make progress in their hepatitis B elimination efforts [1]. If one of the models is found to be superior and more and feasible to implement, it could be considered for broader implementation in other countries in the region.

One strength of the study is the utilization of existing health infrastructure, as we believe this increases sustainability and transferability of the study results. Besides training of staff, the study will utilize existing health structures and staff employed at local clinics/hospitals, to strengthen regional health authorities and ensure local support. Generalizability is increased by having two sites from separate regions of Ethiopia in every treatment model, and by taking a«real-world» pragmatic approach by having broad inclusion criteria and limited exclusion criteria. Key challenges will be to ensure that the six sites are able to complete the necessary screening efforts and reach the predefined sample size. Furthermore, we may expect high rates of loss to follow-up among both treated and untreated participants, as demonstrated by a recently published study from a similar setting [27]. 

A limitation of the study is the observational design. A cluster randomized trial would have ensured comparability of baseline characteristics across the three models, but was not possible with the available budget. Another limitation is the limited granularity in the assessment of deaths, as it is hard to distinguish liver-related deaths from non-liver related deaths in our setting. Lastly, a longer observation time would be preferable to demonstrate differences between the three models in terms of preventing liver failure or death, as liver related complications such as cirrhosis and HCC take time to develop. However, we argue that the effect of TDF on reducing the risk of liver failure or death can be measured within the designated observation period, as outlined in the sample size calculation [20, 28].

In conclusion, this observational cohort study will assess three models of decentralized care for delivering hepatitis B care in rural Ethiopia. From a public health perspective, identifying a simple, feasible and effective model of decentralized CHB care would be a key step to reduce liver-related mortality on the African continent. This study may have important policy implications and is expected to inform national and international hepatitis B elimination efforts in LMICs.

## Data Availability

No datasets are included in this manuscript, but the study protocol and data required to support the study protocol will be supplied on request.

## Abbreviations

ALT: Alanine aminotransferase
ANC: Antenatal care
AST: Aspartate aminotransferase
APRI: Aspartate aminotransferase to platelets ratio
CFIR: Consolidated framework for implementation research
CHB: Chronic hepatitis B
CICI: Context and implementation of complex interventions
CT: Computer tomography
DBS: Dried blood spot
HBV: Hepatitis B virus
HbsAg: Hepatitis B virus surface antigen
HBV DNA: Hepatitis B virus deoxyribonucleic acid
HCC: Hepatocellular carcinoma
HCV: Hepatitis C virus
HIV: Human immunodeficiency virus
LMIC: Low- and middle income country
LSM: Liver stiffness measurement
MRI: Magnetic resonance imaging
NA: Nucleos(t)ide analogue
SD: Standard deviation
TDF: Tenofovir disoproxil fumarate
WHO: World health organization
